# Correction: Transcription factor clusters regulate genes in eukaryotic cells

**DOI:** 10.7554/eLife.45804

**Published:** 2019-02-06

**Authors:** Adam JM Wollman, Sviatlana Shashkova, Erik G Hedlund, Rosmarie Friemann, Stefan Hohmann, Mark C Leake

Wollman AJM, Shashkova S, Hedlund EG, Friemann R, Hohmann S, Leake MC. 2017. Transcription factor clusters regulate genes in eukaryotic cells. *eLife* **6**:e27451. doi: 10.7554/eLife.27451.

Published 25, August 2017

We were recently asked to share strains we used in our study. While checking the strain numbers and strain numbers in our collection, we discovered that the genotype of the strain YSH2856 in Table 1 was provided incorrectly.

Below, the original text of YSH2856 genotype description in Table 1:

"*MATa MIG1-eGFP-KanMX NRD1-mCherry-HphNT1 snf1Δ::LEU2 MET LYS*"

and the corrected version:

"*MATa MIG1-eGFP-HIS3 NRD1-mCherry-HphNT1 snf1Δ::LEU2 MET LYS"*

The strain YSH2856 was developed by providing a *snf1Δ* mutation to the strain YSH2348, therefore, the original two mutations *MIG1-eGFP-HIS3* and *NRD1-mCherry-HphNT1* were unchanged.

The article has been corrected accordingly. We apologise for any inconvenience it might have caused.

