# Supplementary material for: Transcription factor clusters regulate genes in eukaryotic cells
Source: eLife. 2017 Aug 25;6:e27451. doi: 10.7554/eLife.27451 (PMC5602325; doi:10.7554/eLife.27451)
Supplement: Transparent reporting form [file elife-27451-transrepform.docx]

***eLife’s* transparent reporting form**

We encourage authors to provide detailed information *within their submission* to facilitate the interpretation and replication of experiments. If you have any questions, please contact us: [editorial@elifesciences.org](mailto:editorial@elifesciences.org).

**Sample-size estimation**

- You should state whether an appropriate sample size was computed when the study was being designed
- You should state the statistical method of sample size computation and any required assumptions
- If no explicit power analysis was used, you should describe how you decided what sample (replicate) size (number) to use

Please outline where this information can be found within the submission (e.g., page numbers or figure legends), or explain why this information doesn’t apply to your submission:

All sample sizes are stated in each figure caption. The definition of sample size and its justification are described in the Statistical tests and Replicates section of the Methods on p22 line 1004.

**Replicates**

- You should report how often each experiment was performed
- You should include a definition of biological versus technical replication
- The data obtained should be provided and sufficient information should be provided to indicate the number of independent biological and/or technical replicates
- If you encountered any outliers, you should describe how these were handled
- Criteria for exclusion/inclusion of data should be clearly stated
- High-throughput sequence data should be uploaded before submission, with a private link for reviewers provided (these are available from both GEO and ArrayExpress)

Please outline where this information can be found within the submission (e.g., page numbers or figure legends), or explain why this information doesn’t apply to your submission:

Definitions and discussion of biological and technical replicates are described in the Statistical tests and Replicates section of the Methods on p22 line 1004.

**Statistical reporting**

- Statistical analysis methods should be described and justified
- Raw data should be presented in figures whenever informative to do so (typically when N per group is less than 10)
- For each experiment, you should identify the statistical tests used, exact values of N, definitions of center, methods of multiple test correction, and dispersion and precision measures (e.g., mean, median, SD, SEM, confidence intervals; and, for the major substantive results, a measure of effect size (e.g., Pearson's r, Cohen's d)
- Report exact p-values wherever possible alongside the summary statistics and 95% confidence intervals. These should be reported for all key questions and not only when the p-value is less than 0.05.

Statistical tests are discussion in the Statistical tests and Replicates section of the Methods on p22 line 1004. Exact p values are given in the Results section, lines 127, 149, 152; and Figure caption Figure 1 – Figure 1 – Figure Supplement 2 lines 1302, 1303. R^2^ values are given in the Results section lin 331 and again in the figure caption Figure 4, line 1335.

Please outline where this information can be found within the submission (e.g., page numbers or figure legends), or explain why this information doesn’t apply to your submission:

(For large datasets, or papers with a very large number of statistical tests, you may upload a single table file with tests, Ns, etc., with reference to page numbers in the manuscript.)

**Additional data files (“source data”)**

- We encourage you to upload relevant additional data files, such as numerical data that are represented as a graph in a figure, or as a summary table
- Where provided, these should be in the most useful format, and they can be uploaded as “Source data” files linked to a main figure or table
- Include model definition files including the full list of parameters used
- Include code used for data analysis (e.g., R, MatLab)
- Avoid stating that data files are “available upon request”

Please indicate the figures or tables for which source data files have been provided:

As stated in the manuscript, we do no upload additional data files since the data are included in full in multiple summary table. To clarify:

Datasets, we use the 3D 3C model from reference 38:

Z. Duan et al., A three-dimensional model of the yeast genome. Nature. 465, 363–7 (2010).

the model is available as supplementary information from the following link:

<https://www.nature.com/nature/journal/v465/n7296/extref/nature08973-s11.pdb>

the SGD database, now ref 85:

Cherry JM, Hong EL, Amundsen C, Balakrishnan R, Binkley G, Chan ET, Christie KR, Costanzo MC, Dwight SS, Engel SR, Fisk DG, Hirschman JE, Hitz BC, Karra K, Krieger CJ, Miyasato SR, Nash RS, Park J, Skrzypek MS, Simison M, Weng S, Wong ED (2012) Saccharomyces Genome Database: the genomics resource of budding yeast. Nucleic Acids Res. Jan;40(Database issue):D700-5. [PMID:22110037]

We also refer to GFP reporter on addgene:

<https://www.addgene.org/75360/>

It is plasmid number: (Plasmid #75360) and is currently unpublished by Stefan Hohmann

Also, the code is cited as reference (89) to our sourceforge as per your suggestions although we note that sourceforge do not have a commit number function enabled.
